# Important Variations of Aortic Branches: Imaging Case Series

**DOI:** 10.7759/cureus.61901

**Published:** 2024-06-07

**Authors:** Anil K Singh

**Affiliations:** 1 Radiodiagnosis, Sanjay Gandhi Postgraduate Institute of Medical Sciences, Lucknow, IND

**Keywords:** truncus bicaroticus, celiacomesenteric trunk, high riding brachiocephalic artery, aberrant right subclavian artery (arsa), aortic branches

## Abstract

Various anatomical variations are known to occur in branches of the aorta. Some of these variations are common while others are quite uncommon. However, these variations carry significant implications when the patient is diseased and some intervention or surgical procedure is to be done. Most of these variations are usually incidentally detected. This imaging case series illustrates some clinically important variations of aortic branches including branches of the aortic arch and abdominal aorta, with a review of the literature. All cases illustrated here were detected incidentally.

## Introduction

The aorta is the great arterial trunk of the body which supplies blood to all body parts via its branches. These branches arise from different segments of the aorta in the thoracic and abdominal regions [1]. There is a common pattern of origin and course of these branches in the majority of individuals termed as normal pattern. However, in a subset of the population deviations occur in the origin or course of many of the aortic branches. With radiological advances such as computed tomography (CT) angiography and magnetic resonance angiography, these deviations termed “variations,” “aberrations," or “anomalies” are being detected with increasing frequency and mostly incidentally, contrary to older times when these were detected on autopsies or suddenly encountered during surgery [2]. Many of these variations are clinically significant in terms of involvement by disease process or as an interface in the management of disease which needs to be carefully dealt with. A pre-existing knowledge of these variations helps in better treatment planning and thus minimizing the risk of complications and treatment failures [2-4]. In this imaging case series, clinically important variations in aortic branches such as high riding right brachiocephalic artery (BCA), aberrant right subclavian artery, and common celiacomesenteric trunk are being discussed with imaging illustrations and a review of the literature.

## Case presentation

Case 1: "high-riding" brachiocephalic artery

**Figure 1:**
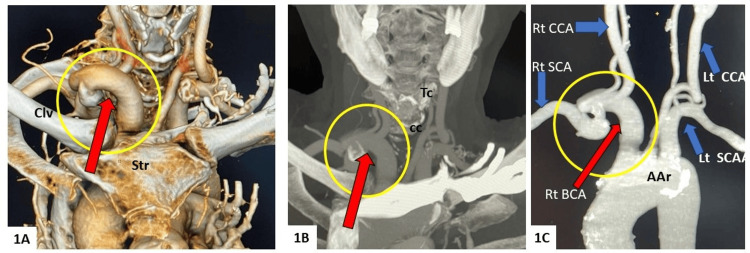
3D volume rendered image (A) and MIP reconstruction from CT angiography study (B and C) showing high riding tortuous right brachiocephalic artery (encircled, red arrow), rising well above suprastrernal notch. C) Bone subtracted maximum intensity projection (MIP) image Clv: clavicle, Str: sternum, Tc: thyroid cartilage, cc: cricoid cartilage, BCA: brachiocephalic artery. SCA: subclavian artery, CCA: common carotid artery, AAr: aortic arch

The case illustrated here refers to incidentally detected high-riding tortuous BCA in an elderly 80-year-old female patient who was being evaluated with a CT angiography study in view of trauma. CT angiography revealed a normal aortic arch branching pattern with the BCA seen to extend and bifurcate above the level of the sternoclavicular joint (~28 mm above the suprasternal notch which corresponds to the level of the sternoclavicular joint) and having a tortuous looped course. The apex of the loop was ~5.0 mm below the level of the cricoid cartilage (Figures 1A-1C). The patient was asymptomatic for this finding.

Case 2: aberrant right subclavian artery with truncus bicaroticus

**Figure 2:**
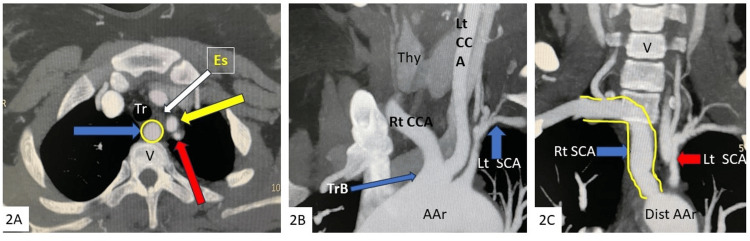
Axial (A) and anterior (B) to posterior (C) coronal MIP images from CT angiography showing aberrant right subclavian artery with prevertebral/retroesophageal course, and having origin from aortic arch distal and posterior to left SCA. Right SCA encircled and indicated by the blue arrow in the axial image (A) and outlined in the coronal image (C). Also noted common trunk origin of the bilateral common carotid artery SCA: subclavian artery; CCA: common carotid artery; Aar: aortic arch; TrB: truncus bicaroticus; Tr: trachea; Es: collapsed esophagus; Thy: thyroid gland, reference for anterior location; V: vertebra, reference for the posterior location

**Figure 3:**
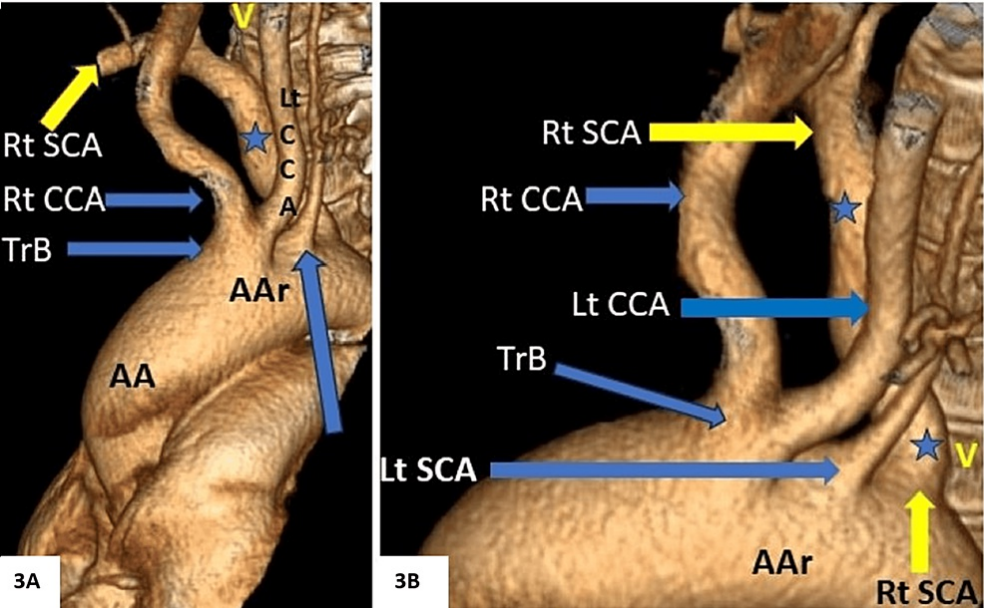
3D volume rendered images from a CT angiography study (A and B) showing aberrant right subclavian artery (Blue Star, annotated) arising as the last branch of the aortic arch, distal and posterior to the left subclavian artery, and traversing to the right side in the prevertebral course AA: ascending aorta; AAr: aortic arch; TrB: truncus bicaroticus; CCA: common carotid artery; SCA: subclavian artery; V: vertebral column

This was a case of Takayasu arteritis in a young female patient who was being evaluated with CT angiography to look for the status of the disease. CT angiography revealed a common carotid trunk (truncus bicaroticus) as the first branch of the aortic arch, with a common trunk dividing into the right and left common carotid artery (CCA). The origin of the common carotid trunk was followed by the origin of the left SCA which was narrowed due to vasculitis. The last and most posterior branch of the aortic arch was the right SCA which was seen originating just distal and posterior to the left SCA and was traversing from left to right side in a retroesophageal course. The retroesophageal segment was displacing the aorta esophagus anteriorly off the vertebral column; however, there was no significant esophageal compression. It was also abutting the trachea (Figures 2A-2C, 3A, 3B). On questioning, the patient did not have overt dysphagia. However, she felt infrequent episodes of feeling hold-up of swallowed contents lasting transiently.

Cases 3-5: common celiacomesenteric trunk

**Figure 4:**
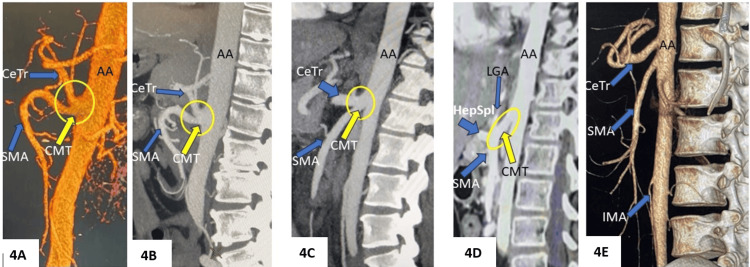
Reformatted images from CT angiography study. A-D showing celiacomesenteric trunk (CMT) in three cases. A and B represent single case, C and D represent two different cases. For comparison, image E showing a more commonly seen "normal" pattern as a separate origin of celiac trunk and SMA. CMT is encircled and indicated by a yellow arrow AA: abdominal aorta; CeTr: celiac trunk; SMA: superior mesenteric artery; HepSpl: hepatosplenic trunk; LGA: left gastric artery; IMA: inferior mesenteric artery

Here are depicted three cases of celiacomesenteric trunk (CMT) in one elderly and two young adult male patients. All these patients underwent multiphasic contrast-enhanced CT study of the abdomen (angiography with enterography) for varied reasons not referrable to this finding. The reasons for CT scans in these patients were recurrent abdominal pain (3), constipation (2), chronic diarrhea (1), and chronic anemia (1). In all three cases, CT studies revealed a single common trunk arising from the abdominal aorta with this common trunk further dividing into the superior mesenteric artery (SMA) and celiac trunk or celiac trunk branches (Figures 4A-4D), instead of the more commonly seen normal pattern of separate origin of celiac trunk and SMA from abdominal aorta (Figure 4E). In two cases, the common trunk of CMT was short <10 mm (Figures 4A-4C), and the other common trunk was 15 mm (Figure 4D). In one case (longer trunk type), the level of origin from the aorta corresponded to the L1-L2 intervertebral disc, and in the other two cases with a short trunk level of origin was the upper margin of the L1 vertebral body. In all cases, arteries were patent with normal caliber (Figures 4A-4D). In the case with a longer trunk, the angle and distance with the abdominal aorta were in the lower range of ~16^o^ and 8 mm, respectively, but this patient did not have any relevant symptoms such as postprandial abdominal fullness and vomiting. His main complaints were constipation and recurrent lower abdominal pain. In two other patients, these angle and distance measurements were within normal limits.

## Discussion

High-riding BCA

The BCA is the first branch of the aortic arch and it branches into the right SCA and CCA, usually at the thoracic inlet level. High-riding BCA refers to the extension of BCA into the lower neck where it may go unnoticed by the patient or present as a pulsatile lump [4,5].

Normally, it courses superiorly and to the right of the thoracic trachea and divides into the right subclavian and right common carotid arteries behind the sternoclavicular joint, at the level of the suprasternal notch. This normal pattern can be seen in up to ~70% of individuals. Embryologically, the BCA develops from two components i.e., aortic sac and proximal right fourth aortic arch. High riding brachiocephalic trunk results from the persistence of a part of the proximal segment of the right fourth aortic arch leading to elongation of the brachiocephalic trunk superiorly above the level of sternoclavicular joint (suprasternal notch) [6,7].

The BCA is classified as high-lying (prolonged) if it bifurcates superior to the sternoclavicular junction. The prevalence of this anatomical variation reported by radiological studies varies from 3% to 26.4% [6,7]. High-riding BCA can go as high as the level of cricoid and thyroid cartilage, hence overlapping with the thyroid gland [6,7]. Its presence has been correlated with potential complications such as fistulas and bleeding with surgeries and interventional procedures in the neck including thyroid surgery, mediastinoscopy, percutaneous tracheotomy, and placement of central venous catheters [6-11]. Cases of hemorrhage and fistulas in neck surgeries such as thyroidectomy and tracheostomy have been attributed to high-riding BCA [12-14].

For this variation, different prevalences have been reported from different geographical areas. In the study by Jasso-Ramírez et al. in the Mexican population high-lying BCA was seen in as high as 64.81% [6]. In the study by Cai et al., in the Chinese population, the prevalence of this variation was seen in 26.4% of cases. However, the upper edge of BCA > 2.0 cm above the suprasternal notch was seen in only 2.2% of cases, and they reported higher surgical risks in this subgroup [7]. In a study by Weightman et al., involving the Australian population, bifurcation of the BCA above the suprasternal notch was reported in 38.2% of cases [15].

Identifying this variation is necessary to alleviate apprehension in otherwise healthy patients with lower neck lumps. Also, it is important to keep it in knowledge so as to plan surgery or vascular intervention if any in relevant anatomical locations. This will avoid inadvertent injury to the vessel. This anomaly also causes difficulty in the cannulation of the right CCA and SCA during the endovascular intervention.

This can be detected on routine ultrasound of the neck or other imaging modalities such as contrast-enhanced computed tomography (CECT) or MRI. If detected on USG, it should be evaluated with CECT thorax to rule out other causes of upward displacement of the artery. Until recently, it was considered an uncommon variation.

Aberrant right SCA

Usually, the right SCA arises from the BCA which itself arises as the first branch of the aortic arch. Aberrant right SCA takes its origin as the last and most posterior branch of the aortic arch, distal to the origin of the left SCA, and traverses from the left to the right side in a retroesophageal course. The patient may present with symptoms of difficulties in swallowing. Aberrant right SCA is also known as “arteria lusoria” because of the dysphagia caused by it. It is considered to be the most common embryological abnormality of the aortic arch [16,17]. The prevalence of this variation has been reported to be 0.5% to 2.5% [18].

Aberrant right SCA may be also accompanied by Kommerell’s diverticulum at its origin [16]. In cases with aberrant right SCA, there is no common brachiocephalic trunk. Right CCA arises directly from the aortic arch as its first branch or sometimes it may arise via a common trunk which also gives off left CCA, and the common trunk is known as “truncus bicaroticus” [19]. In the study by Klinkhamer which analyzed 295 patients, truncus bicaroticus was seen in 29% of patients [20].

In the majority of cases, aberrant right SCA is asymptomatic. However, in some individuals, it can cause pressure symptoms such as dysphagia, dyspnea, and stridor. Dysphagia has been reported to occur in 7% to 8% of adults with this anomaly [20-22]. Aneurysms of the aberrant right SCA have been also reported. Patients with aneurysm are more likely to be symptomatic [21,23]. If in symptomatic cases, endoscopy reveals extrinsic compression, the possibility of aberrant vessel should be also kept in mind and the patient should be evaluated with cross-sectional imaging such as contrast-enhanced CT or MRI to confirm it or find an alternative diagnosis.

Aberrant SCA poses risks in thoracic surgeries such as esophagectomy. If detected on imaging workup for any indication it needs to be reported. It becomes even more important if the patient has to undergo some thoracic surgery or endoscopic intervention. A beforehand knowledge of its existence will help in tailored and cautious retroesophageal dissection while proceeding with esophagectomy, thus avoiding the risk of arterial injury [17,24-26].

This anomaly needs to be kept in mind while performing endoscopic interventions or when esophageal or posterior mediastinal surgery is to be performed, to avoid injury to this artery. A pre-existing knowledge of its existence also facilitates endovascular interventions, which otherwise may take a relatively longer time to search and cannulate this vessel.

Common celiacomesenteric trunk

It refers to the origin of the celiac trunk and SMA via a common trunk from the abdominal aorta. The celiac trunk usually arises at the level of the 12th thoracic vertebra and gives off the left gastric artery, the common hepatic artery, and the splenic artery, in some cases inferior phrenic arteries. The SMA arises ~1 cm below the origin of the celiac trunk (at the level of the L1 vertebra). In embryonic development, the abdominal visceral arteries arise from the primitive dorsal abdominal aorta through four roots: the left gastric artery, the hepatic artery, the splenic artery, and the SMA. These roots are joined together by a longitudinal ventral anastomosis. Normally, a cleft forms in this anastomosis between the third and fourth roots with the separation of the celiac trunk from SMA (Figure 3E). Failure of this separation results in a common CMT which is a rare variation of the anterior branches of the abdominal aorta. The overall reported incidence is less than 5% across various studies. Some studies have reported an incidence as low as 0.5% [27-29]. In a study by Yan et al., it was 2.38% [30].

Variations in incidence have also been found to be related to geographical areas and in some cases gender [31,32]. Celiac trunk variations have been reported to be more common in Japanese and Korean populations than in Caucasian ones [32].

Morita et al. classified CMTs into 4 major types and a total of 10 subtypes. Type I is the common trunk of all three branches of CA with SMA (classic type); type II is the common trunk of any two branches of CA with SMA and the third arising directly from the aorta; type III is the common trunk of any one branch of CA with SMA and other two branches having independent aortic origin; type IV is the common trunk of any one branch of CA with SMA and other two branches having common origin from aorta [33,34].

Tang et al. proposed a five-type classification system on the basis of CT angiography studies. They also took into consideration the direct separate origin of one of the celiac branches from the abdominal aorta. Type I is all branches of the celiac trunk arising from CMT (hepato-gastro-spleno-mesenteric trunk). Type II is a hepato-spleno-mesenteric trunk with LGA arising from the aorta. Type III is a gastro-spleno-mesenteric trunk with CHA arising from the aorta. Type IV is a hepato-gastro-mesenteric trunk with SA arising from the aorta. Type V is any other variation that meets the above definition of the CMT. Type I was considered a complete type in view of all branches arising via the common trunk; type II-V was considered incomplete in view of one of the celiac trunk branches arising directly from the aorta [28].

CMT has been also classified into short and long trunk types depending on the length of the common trunk. In the study by Tang et al., short trunks ranged from 6 mm to 14 mm, while long trunks ranged from 17 mm to 39 mm [28]. However, no cut-off point for short and long trunks could be found in the literature. In the cases illustrated here in this series, the common trunk was less than 10 mm in two cases and ~15 mm in one case.

This anatomical variation is usually asymptomatic and may be discovered incidentally during imaging studies (USG Doppler, CECT, contrast-enhanced magnetic resonance imaging (CEMRI)), vascular explorations, or during a cadaver’s dissection. Rarely, it may cause symptoms including non-specific abdominal pain to abdominal angina when associated with atherosclerosis. The presence of a common trunk proves disadvantageous in terms of common afflictions of two arterial trunks by a single lesion. In the case of a separate celiac trunk and SMA, even if one artery is stenosed or dissected, other arteries along with IMA are sufficient to maintain the circulation of the bowel. This advantage is lost in cases with a common trunk [28,35-37].

CMT is a rare anatomical variation, putting the patient at a potentially disadvantageous status in terms of a disease such as a stenosis, aneurysm, or dissection affecting both celiac and SMA components simultaneously, and thus precluding possible sparing of one of the arteries which might be the case when arteries arise separately. It is also important from a surgical or intervention point of view. Extra precaution and effort are needed to work with and protect or preserve this dominant arterial trunk of the bowel and hepato-biliary-pancreatic system.

## Conclusions

Many variations occur in aortic branches, some of these having important clinical implications. Above mentioned variations including high-riding BCA, aberrant right SCA, and common celiaco-mesenteric trunk are clinically important. Though most of the time these are detected incidentally, these must be reported if detected. Also, their presence can be enquired on imaging studies if some intervention or surgical procedure is contemplated in a relevant anatomical area.
